# Bacterial colonization of the peri-implant sulcus in dentate patients: a prospective observational study

**DOI:** 10.1007/s00784-016-1941-x

**Published:** 2016-08-24

**Authors:** M. A. Stokman, A. J. van Winkelhoff, A. Vissink, F. K. L. Spijkervet, G. M. Raghoebar

**Affiliations:** 1 Department of Oral and Maxillofacial Surgery, University of Groningen, University Medical Center Groningen, P.O. Box 30.001, 9700 RB Groningen, The Netherlands; 2 Department of Medical Microbiology, University of Groningen, University Medical Center Groningen, Groningen, The Netherlands; 3 Department of Center for Dentistry and Oral Hygiene, University of Groningen, University Medical Center Groningen, Groningen, The Netherlands

**Keywords:** Bacteria, Colonization, Dental implants, Smoking

## Abstract

**Objectives:**

The aim of the present study was to compare the composition of the periodontal microflora at baseline (T0) with the submucosal microflora at least 1 year after implant placement (T1) in periodontally healthy patients.

**Material and methods:**

For all 169 consecutive patients that visited our clinic during 1 year, we determined their periodontal parameters, implant mucosal index, and presence of implant calculus. At T0, self-reported smoking status was recorded and subgingival and submucosal biofilm samples were obtained and analyzed for the presence and numbers of selected periodontal pathogens. All measurements were repeated at T1.

**Results:**

One hundred twenty patients completed the study. Periodontal parameters were stable or had improved at T1. The total bacterial load was lower at implant sites (*P* < 0.05). The prevalence of *Porphyromonas gingivalis* was low at baseline, but at T1, detection rate and numbers were higher at implant sites compared to dentate sites. At T1, the frequency of detection of *P. gingivalis* (*P* = 0.01), *Parvimonas micra* (*P* = 0.018), and *Fusobacterium nucleatum* (*P* = 0.035) was higher in smoking patients (*n* = 23) than in non-smokers (*n* = 97).

**Conclusions:**

Colonization of the submucosal peri-implant area is similar to the composition of subgingival microbiota. Smoking has a measurable effect on the colonization of implant-associated biofilms and may select for *P. gingivalis*, *P. micra*, and *F. nucleatum*.

**Clinical relevance:**

The colonization of implants by well-known periodontal pathogens is very similar to that in normal dentition, also in a healthy cohort. Smoking status was related with the prevalence of periodontal pathogens where smokers harbored more often periodontal pathogens such as *P. gingivalis*, *P. micra*, and *F. nucleatum*.

## Introduction

Dental implants are used to replace missing teeth and to support crowns, bridges, and prostheses. Dental implants have a high survival rate, and implant therapy is considered highly successful [1, 2]. However, implant-associated infections also occur regularly. Peri-implant mucositis after 10 years is estimated to affect 63 % of patients and 31 % of implants, while peri-implantitis affects 19 % of patients and 10 % of implants [3]. Among other factors, bacteria are thought to play an essential role in both peri-implant mucositis and peri-implantitis [4].

Colonization of the submucosal peri-implant area starts immediately after installation of the implant or the abutment [5]. In edentulous patients, facultative anaerobic streptococci dominate initially [6], followed by facultatively anaerobic rods and gram-negative strict anaerobic rods such as *Fusobacterium* and *Prevotella* species [7]. Using a DNA-DNA hybridization checkerboard technique, Quirynen et al. [8] studied early colonization of dental implants in dentate patients with a history of periodontitis. They observed that periodontitis-associated bacteria of the red cluster, i.e., *Porphyromonas gingivalis*, *Tannerella forsythia*, and *Treponema denticola*, could be detected in the peri-implant sulcus within 1 week after abutment connection. These red complex bacteria were also detected in a significant number of peri-implant sites by Fürst et al. [5]. In patients with a history of periodontitis, *P. gingivalis* could be detected in the peri-implant sulcus 1 month after abutment connection [9]. Takanashi et al. [10] studied colonization of dental implants in patients without a history of periodontitis and demonstrated that *P. gingivalis* and *Prevotella intermedia* are intra-orally transmitted from dentate to peri-implant sites. De Boever and De Boever [11] studied early colonization of non-submerged dental implants in patients with a history of aggressive periodontitis and found no or minor differences between the composition of the dentate and the peri-implant microflora after 6 months in most but not all patients. Van Brakel et al. [12] investigated the early colonization around zirconia and titanium abutments and found no significant differences 3 months post-surgery. Factors that may influence the colonization of the submucosal peri-implant microflora include the presence of natural teeth and the periodontal condition.

Most of the studies summarized above had a limited number of subjects, and these subjects were often patients with a history of periodontitis. Furthermore, most of these studies focused on early colonization. Therefore, the aim of the present study was to compare the composition of the periodontal microflora at baseline with the submucosal microflora at least 1 year after implant placement in periodontally healthy patients.

## Material and methods

### Patients

During 1 year, all consecutive eligible patients who were referred to the Department of Oral and Maxillofacial Surgery of the University Medical Center Groningen (UMCG) for dental implant treatment were included in this observational study. Dentate patients with pockets <6 mm were eligible unless they presented with systemic diseases or had been subjected to head and neck cancer treatment. The study design involved clinical, radiographic, and microbiological examination of the teeth at baseline (T0) and after at least 1 year after implantation (T1), including the peri-implant conditions.

The study was performed in accordance with Dutch law on ethical rules and principles for human research and in accordance with the 1964 Helsinki Declaration. The Medical Ethic Committee of the UMCG agreed with the study protocol (M15.184424).

### Clinical parameters

At T0, periodontal measurements were taken at six sites per tooth (mesiobuccal, mesiolingual, distobuccal, distolingual, mid-buccal, and mid-lingual) using a manual probe. The clinical periodontal parameters included probing depth, modified plaque index (mPlI) (0 = no plaque, 1 = plaque on the probe, 2 = plaque seen by the naked eye, 3 = abundance of soft matter) [13], modified sulcus bleeding index (mBI) (0 = no bleeding, 1 = isolated bleeding spots, 2 = confluent line of blood, 3 = heavy or profuse bleeding) [13], recession (measured from the gingival margin to the cementoenamel junction (CEJ); 0 = gingival margin was located coronal to the CEJ, 1 = gingival margin located apical to CEJ), and the absence (0) or presence (1) of suppuration. At T1, the same periodontal parameters were determined for the teeth and the implants. Additionally, for the implants, the implant mucosal index [14] and the absence (0) or presence (1) of calculus were determined. The self-reported current smoking status was recorded at T0 and T1.

### Microbiological analysis

At baseline, subgingival samples were taken from the deepest and/or bleeding pocket in each quadrant of the dentition. If a patient had no signs of periodontal disease (pockets <4 mm, no bleeding on probing), the samples were taken from the mesiopalatinal pocket of the first molars. If the first molars were not present, the second premolar was selected. If a patient had two or more implants, the samples from implants were pooled. At T1, this procedure was repeated at the same sample sites and peri-implant samples were taken. Two sterile paper points per tooth/implant were inserted to the depth of the pockets and left in place for 10 s and were collected and pooled in 2 ml reduced transport fluid [15]. The samples were processed for microbiological examination within 1 h after sampling.

The microbiological samples were analyzed according standard anaerobe culture techniques for the presence and numbers of *Aggregatibacter actinomycetemcomitans*, *P. gingivalis*, *P. intermedia*, *T. forsythia*, *Parvimonas micra*, *Fusobacterium nucleatum*, and *Campylobacter rectus* [16]. Also, the total number of colony-forming units per sample was determined [17, 18].

### Statistical analysis

Changes over time for dichotomous data were analyzed with McNemar’s test. For ordinal data, the Wilcoxon signed-rank test was used. Differences between groups were analyzed with the Mann-Whitney test. A sub-analysis was performed between the patients with a single tooth replacement and an overdenture. Two-sided *P* values <0.05 were considered statistically significant.

Multiple logistic regression analysis was used with the following variables to determine their predicted value influencing periodontal bacterial species at the follow-up assessment: age, smoking at follow-up assessment, use of antibiotics at baseline, modified plaque index at the implant site, modified sulcus bleeding index at the implant site, location of the implant (anterior or posterior), pocket depth at implant site, use of antibiotics at follow-up assessment, and presence of the periodontal bacteria at baseline. The variables that were significantly associated with the outcome variable (*P* ≤ 0.10) were entered in the logistic regression analyses. Thereafter, variables not significantly contributing to the regression equation were removed (*P* > 0.10). All data were analyzed using IBM SPSS Statistics 22.

## Results

**Fig. 1:**
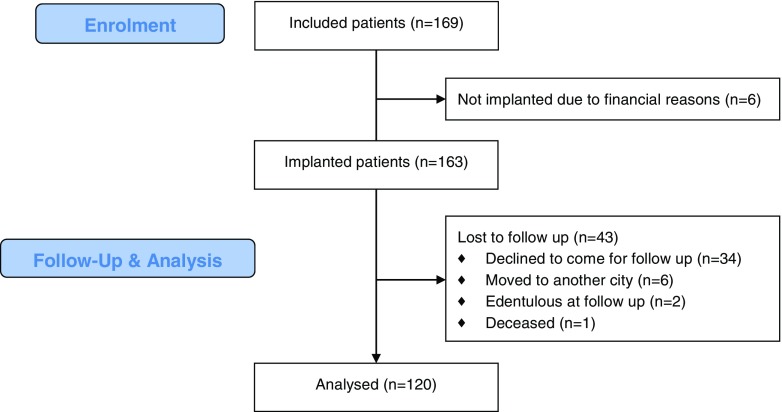
Flowchart of patient recruitment for the study

One hundred sixty-nine consecutive eligible patients were included in this observational study: 83 males (43.6 ± 16.9 years, range 18–74 years) and 86 females (47.3 ± 16.3 years, range 18–79 years). Of these 169 patients, 6 did eventually not receive implants and 43 were lost to follow-up for various reasons (Fig. 1). Consequently, 120 patients remained for final analysis. The demographic parameters are presented in Table 1. No significant differences in baseline variables were observed between the total group of 169 patients and the 120 patients that completed follow-up. The mean time between implantation and follow-up was 17 ± 3 months.

**Table 1:** Characteristics of the analyzed patients (*n* = 120) and the total number of eligible patients (*n* = 169) at baseline

Patients’ characteristics	120, *n* (%)	169, *n* (%)
Age mean ± SD (years)	46.3 (16.5)	45.5 (16.7)
Gender: male/female	59/61	83/86
Type of reconstruction		
Single tooth replacement	106 (88.3)	NA
Overdenture maxilla	13 (10.8)	NA
Overdenture mandibula	1 (0.8)	NA
Number of implants per patient		
1	62 (51.7)	87 (51.5)
2	33 (27.5)	40 (23.7)
3	4 (3.3)	6 (3.6)
4	7 (5.8)	10 (5.9)
5	3 (2.5)	4 (2.4)
6	9 (7.5)	13 (7.7)
7	1 (0.8)	2 (1.2)
8	1 (0.8)	1 (0.6)
Missing	0 (0)	6 (3.6)
Type of implant		
Astra	4 (3.3)	4 (2.4)
Bone Level Roxolid	1 (0.8)	2 (1.2)
Bone Level Straumann	50 (41.7)	70 (41.4)
Brånemark	3 (2.5)	6 (3.6)
3i	7 (5.8)	8 (4.7)
NobelActive	2 (1.7)	2 (1.2)
NobelReplace	9 (7.5)	12 (7.1)
NobelSpeedy	1 (0.8)	2 (1.2)
Standard Straumann	16 (13.3)	20 (11.8)
Standard plus Straumann	24 (20.0)	33 (19.5)
Combination of types	3 (2.5)	4 (2.4)
Missing	0 (0)	6 (3.6)
Augmentation		
No	55 (45.8)	76 (45.0)
Yes, before implantation	47 (39.2)	63 (37.3)
Yes, during sinus augmentation	3 (2.5)	3 (1.8)
Yes, during implantation	15 (12.5)	21 (12.4)
Missing	0 (0)	6 (3.6)
Implant location		
Front	34 (28.3)	46 (27.2)
Lateral parts	72 (60.0)	97 (57.4)
Front and lateral parts	14 (11.7)	20 (11.8)
Use of antibiotics during the last 3 months		
No	88 (73.3)	124 (73.4)
Yes	32 (26.7)	45 (26.6)
Reason of antibiotics use		
Not applicable	88 (73.3)	124 (73.4)
Augmentation	23 (19.2)	32 (18.9)
Others	9 (7.5)	13 (7.7)
Self-reported smoking		
No	93 (77.5)	124 (73.4)
Yes	27 (22.5)	45 (26.6)

No significant differences were present between the analyzed patients and the total group

*NA* not assessed

### Clinical parameters

The clinical periodontal parameters at T0 and T1 and clinical parameters at the implant sites at T1 are shown in Table 2. At T0, 97.5 % of the patients showed maximum probing pocket depth ≤4 mm; at T1, this was 96.3 %. Compared to T0, significantly less plaque accumulation was observed at follow-up (*P* = 0.001). All other recorded periodontal parameters showed no statistically significant changes between the T0 and T1 for the teeth. At T1, the maximum probing pocket depth ≤4 mm at the implant sites was 94.1 %. The mPlI was significantly lower at the implant sites at T1 compared to T0 at the teeth (*P* < 0.01). In contrast, the mBI at the implant sites was significantly higher compared to the teeth at T0 and T1 (respectively, *P* = 0.009 and *P* = 0.002); this higher mBI predominantly referred to isolated bleeding spots.

**Table 2:** Clinical periodontal and peri-implant parameters at T0 and T1

Clinical parameters	Baseline (T0)	Follow-up (T1)	
	Teeth, *n* (%)	Teeth, *n* (%)	Implants, *n* (%)
Self-reported smoking			
No	93 (77.5)	97 (80.8)	97 (80.8)
Yes	27 (22.5)	23 (19.2)	23 (19.2)
Modified plaque index			
Score 0, no detection of plaque	70 (58.3)	89 (74.2)	104 (86.7)
Score 1, plaque on the probe	35 (29.2)	23 (19.2)	9 (7.5)
Score 2, plaque seen by the naked eye	15 (12.5)	8 (6.7)	4 (3.3)
Score 3, abundance of soft matter	0 (0)	0 (0)	3 (2.5)
Deepest pocket (mm)			
1	0 (0)	0 (0)	1 (0.8)
2	42 (35.0)	44 (36.7)	58 (48.3)
3	57 (47.5)	60 (50.0)	42 (35.0)
4	18 (15.0)	12 (10.0)	12 (10.0)
5	3 (2.5)	4 (3.3)	3 (2.5)
6	0 (0)	0 (0)	3 (2.5)
10	0 (0)	0 (0)	1 (0.8)
Modified sulcus bleeding index			
Score 0, no bleeding	91 (75.8)	89 (74.2)	67 (55.8)
Score 1, isolated bleeding spots	22 (18.3)	28 (23.3)	44 (36.7)
Score 2, confluent line of blood	7 (5.8)	3 (2.5)	9 (7.5)
Score 3, heavy or profuse bleeding	0 (0)	0 (0)	0 (0)
Implant mucosal index			
Score 0, normal mucosa	NA	NA	80 (66.7)
Score 1, mild inflammation	NA	NA	36 (30.0)
Score 2, moderate inflammation	NA	NA	4 (3.3)
Score 3, severe inflammation	NA	NA	0 (0)
Implant dental calculus present			
Score 0, no dental calculus	NA	NA	118 (98.3)
Score 1, dental calculus present	NA	NA	2 (1.7)
Recessions			
No	100 (83.3)	97 (80.8)	119 (99.2)
Yes	20 (16.7)	23 (19.2)	1 (0.8)
Suppuration			
No	120 (100)	120 (100)	120 (100)
Yes	0 (0)	0 (0)	0 (0)

*NA* not assessed

### Smoking

At baseline, the self-reported current smoking status identified 93 non-smokers (77.5 %) and 27 smokers (22.5 %). At T1, 4 patients had stopped smoking, resulting in 97 non-smokers and 23 smokers (Tables 1 and 2). No significant differences were found between the non-smoking and smoking groups for any of the clinical periodontal parameters.

### Microbiological analysis

**Fig. 2:**
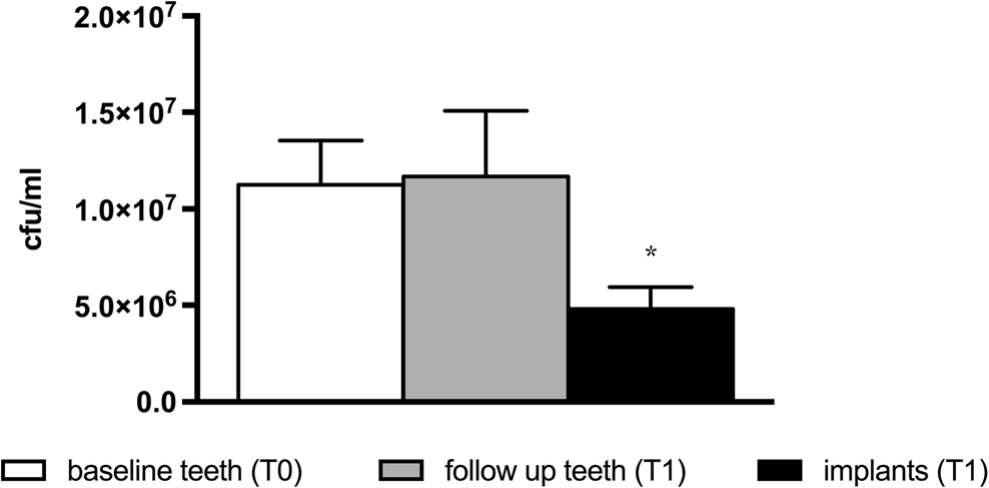
Total mean bacterial load (cfu/ml) ± SEM (*N* = 126). *Asterisk* significantly different from T0 and T1 periodontal values

The mean total bacterial load (colony-forming units (cfu)/ml) at the dentate sites did not differ between T0 and T1 and was significantly higher than that at the implant sites at T1 (1.13*E* + 07 vs 4.8*E* + 06) (Fig. 2; *P* < 0.05). A sub-analysis between single tooth replacements and overdentures showed that the mean total bacterial load (cfu/ml) in the overdenture group was significantly higher at the implant site at T1 compared to T0 at the dentate sites. This was probably caused by a higher mBI. However, this subgroup of patients with an overdenture was very small, only 14 patients.

**Fig. 3:**
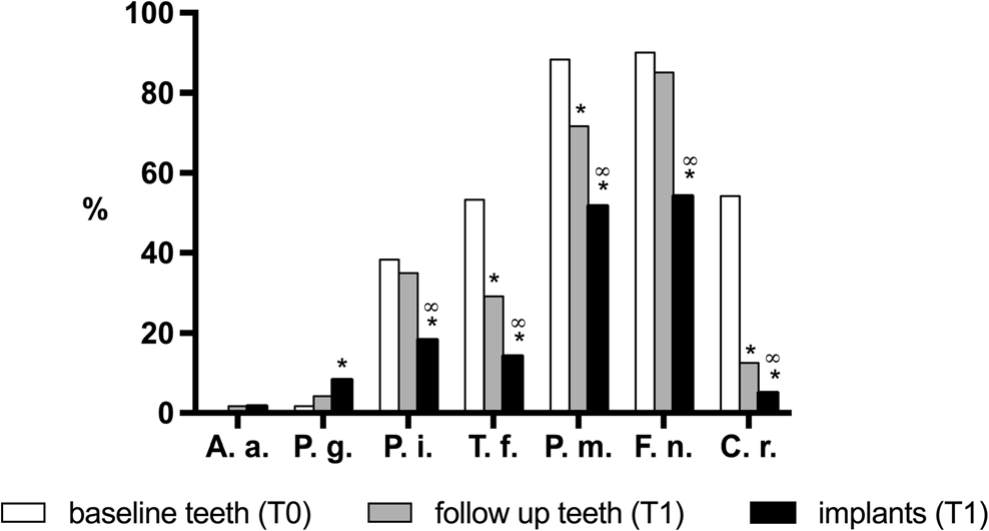
Prevalence (%) of selected periodontal pathogens. *Asterisk* significantly different from T0 values; *infinity* significantly different from T1 values. *A.a. Aggregatibacter actinomycetemcomitans*, *P.g. Porphyromonas gingivalis*, *P.i. Prevotella intermedia*, *T.f. Tannerella forsythia*, *F.n. Fusobacterium nucleatum*, *P.m. Parvimonas micra*, *C.r. Campylobacter rectus*

The prevalence of selected periodontal bacterial species at dentate sites at T0 and T1 is depicted in Fig. 3. The prevalence of *A. actinomycetemcomitans* was <2 % in all three groups. At the dentate sites, the prevalence of *T. forsythia*, *P. micra*, and *C. rectus* was significantly lower at T1 compared to T0 (*P* < 0.01). At implant sites, the prevalence of *P. intermedia*, *T. forsythia*, *P. micra*, *F. nucleatum*, and *C. rectus* species was significantly lower compared to the teeth at T0 and T1 (*P* < 0.05). In contrast, the prevalence of *P. gingivalis* had increased at T1 at dentate sites and was higher at the implant sites (*P* = 0.039).

**Fig. 4:**
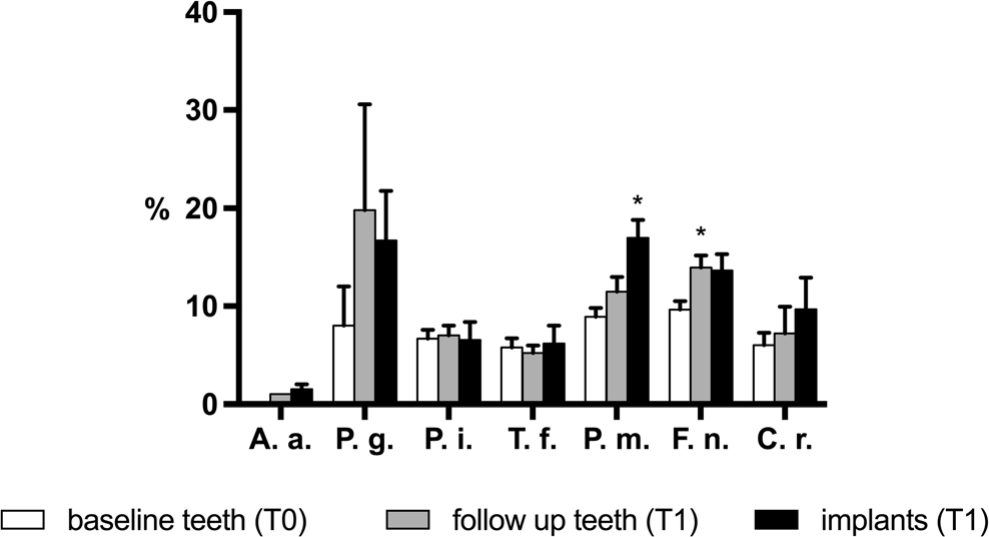
Relative abundance of selected periodontal pathogens ± SEM in culture-positive patients. *Asterisk* significantly different from T0 values. *A.a. Aggregatibacter actinomycetemcomitans*, *P.g. Porphyromonas gingivalis*, *P.i. Prevotella intermedia*, *T.f. Tannerella forsythia*, *F.n. Fusobacterium nucleatum*, *P.m. Parvimonas micra*, *C.r. Campylobacter rectus*

The proportions of selected periodontal pathogens in culture-positive patients are depicted in Fig. 4. At dentate sites, a higher mean percentage at T1 compared to T0 was observed for *P. gingivalis*, *P. intermedia*, *P. micra*, *F. nucleatum*, and *C. rectus*, but the differences were significantly higher only for *F. nucleatum* (*P* = 0.005). At implant sites, the mean percentage of *A. actinomycetemcomitans*, *T. forsythia*, *P. micra*, and *C. rectus* was higher compared to the dentate sites at T1, but the differences were not significant. Comparing the implant sites with the dentate sites at T0, a significantly higher mean was observed only for *P. micra* at the implant sites (*P* < 0.001).

### Effect of smoking on the dentate and implant microflora

**Fig. 5:**
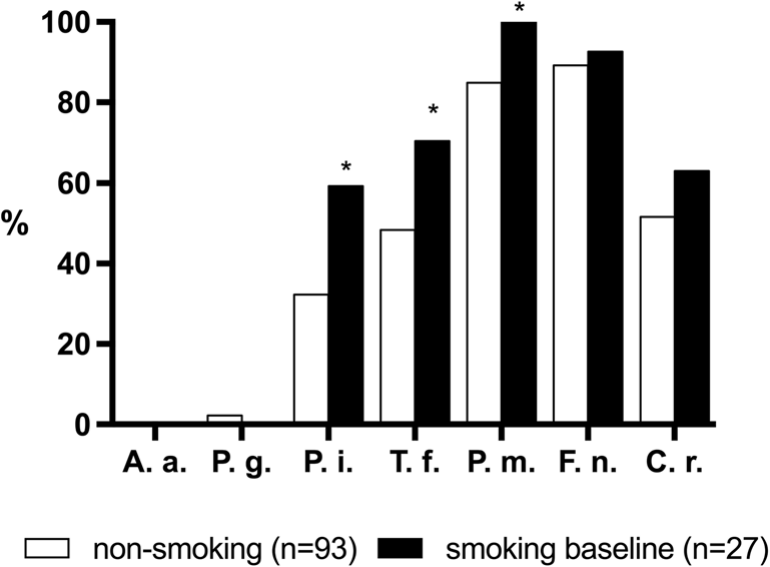
Prevalence (%) of selected periodontal pathogens in smokers (*n* = 30) and non-smokers (*n* = 96) at T0. *Asterisk* significant difference between both groups. *A.a. Aggregatibacter actinomycetemcomitans*, *P.g. Porphyromonas gingivalis*, *P.i. Prevotella intermedia*, *T.f. Tannerella forsythia*, *F.n. Fusobacterium nucleatum*, *P.m. Parvimonas micra*, *C.r. Campylobacter rectus*

At baseline, the prevalence of *P. intermedia*, *T. forsythia*, *P. micra*, *F. nucleatum*, and *C. rectus* at dentate sites was higher in the smoking group (*n* = 27) compared to the non-smoker group (*n* = 93) with statistically significant differences for *P. intermedia* (*P* = 0.011), *T. forsythia* (*P* = 0.045), and *P. micra* (*P* = 0.033) (Fig. 5).

**Fig. 6:**
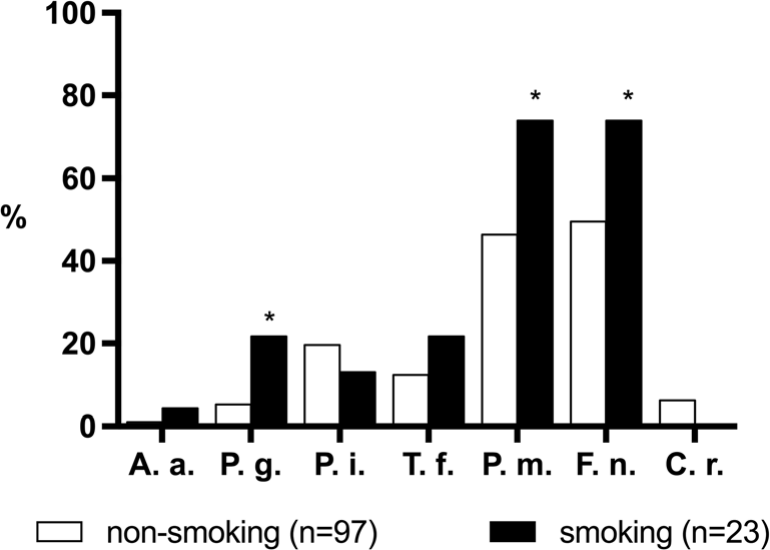
Prevalence (%) of selected periodontal pathogens at implants in smokers (*n* = 26) and non-smokers (*n* = 100) at T1. *Asterisk* significant difference between both groups. *A.a. Aggregatibacter actinomycetemcomitans*, *P.g. Porphyromonas gingivalis*, *P.i. Prevotella intermedia*, *T.f. Tannerella forsythia*, *F.n. Fusobacterium nucleatum*, *P.m. Parvimonas micra*, *C.r. Campylobacter rectus*

The prevalence of the selected bacterial species in the peri-implant microflora at T1 also showed differences between smokers and non-smokers with a significantly higher prevalence in smokers for *P. gingivalis* (*P* = 0.01), *P. micra* (*P* = 0.018), and *F. nucleatum* (*P* = 0.035) (Fig. 6).

The proportions of selected periodontal pathogens in culture-positive patients in the dentate sites at T0 and T1 and at implant sites were not significantly different between smokers and non-smokers.

### Multiple logistic regression analysis

The results of the multiple logistic regression analysis of the various parameters and their predicted value influencing periodontal bacterial species at the follow-up assessment are presented in Table 3. *P. gingivalis*, *P. micra*, and *F. nucleatum* were more often seen in smokers, *P. intermedia* more often in subjects with a higher modified sulcus bleeding index, *T. forsythia* and *P. micra* more often in subjects with deeper pockets, and *F. nucleatum* in subjects with a higher modified plaque index.

**Table 3:** Significant multivariable associations (*P* < 0.10) with the presence of periodontal pathogens at implant sites at the follow-up measurement

Periodontal pathogen	Variable	Multiple regression analysis				
		*B* ^a^	S.E.	OR	95 % CI	*P*
*Aggregatibacter actinomycetemcomitans*	–					
*Porphyromonas gingivalis*	Smoking	1.63	0.68	5.11	1.34–19.49	0.02
*Prevotella intermedia*	Modified sulcus bleeding index	−0.90	0.52	0.41	0.15–1.13	0.08
*Tannerella forsythia*	Pocket depth	0.61	0.23	1.83	1.17–2.87	0.01
*Parvimonas micra*	Smoking	1.17	0.53	3.22	1.14–9.12	0.03
	Pocket depth	0.38	0.21	1.47	0.98–2.20	0.07
*Fusobacterium nucleatum*	Smoking	0.98	0.53	2.67	0.95–7.50	0.06
	Modified plaque Index	1.39	0.68	4.00	1.06–15.10	0.04
*Campylobacter rectus*	Antibiotic use at baseline	1.82	0.89	6.14	1.07–35.35	0.04

*S.E.* standard error, *OR* odds ratio, *95 % CI* 95 % confidence interval

^a^Regression coefficient

## Discussion

In this study, we assessed the microflora of the peri-implant sulcus by bacterial species that are associated with progression of periodontal disease [16] and peri-implantitis [19] in dentate patients with minimal periodontal inflammation at baseline. Colonization of the submucosal peri-implant area is similar to the composition of the subgingival microbiota, but the total bacterial load is significantly lower in the implants compared to the teeth. Previous studies have shown a similar composition of the microflora between teeth and implants on the short term [5, 10, 11]. Furthermore, in our prospective observational study, the periodontal parameters were assessed at baseline while in many studies, the observations were retrospective and baseline measurements were not available [20].

Although the probing pocket depth distribution was comparable between dentate and implant sites, the total cultivable bacterial load (cfu/ml) was significantly lower at the implant sites. This may be related to the low plaque index at the implant sites: 87 % of the patients had a mPlI of 0. This does not explain the significantly higher modified sulcus bleeding index at the implants compared to the teeth, although this increase predominantly concerned isolated bleeding spots. This could be explained by the difference in the composition of the connective tissue, the alignment of the collagen bundles, and the distribution of vascular structures in the compartment apical of the junctional epithelium between the gingiva at teeth and the mucosa at implants [21].

The prevalence of two major periodontal pathogens, *A. actinomycetemcomitans* and *P. gingivalis*, was low at T0 and T1, which reflects the healthy periodontal condition of the study subjects [22].

We found that the prevalence of most of the selected bacterial species at dentate sites had decreased at T1 relative to T0 values and was lowest at implant sites at T1. An exception was the detection of *P. gingivalis*, which had increased at T1 at dentate sites and was highest at implant sites at T1. This could indicate that placement of dental implants may favor the formation of a submucosal biofilm that supports colonization by this pathogen and may explain the frequent detection of this pathogen in peri-implantitis lesions [23]. The highest prevalence of *P. gingivalis* was found at implant sites at T1. We observed a significantly higher proportion of *P. micra* at implant sites relative to dentate sites. This finding is accordance with the recent observation of Eick et al. [20], who also reported a higher prevalence and higher numbers of this species at implant sites.

Our study confirms that smoking significantly affects the composition of the dentate and peri-implant microflora [18, 20, 24]. At baseline, the prevalence of the selected species was higher in current smokers, except for *P. gingivalis* and *A. actinomycetemcomitans*, which is in agreement with earlier findings [18]. Differences in prevalence between current smokers and non-smokers at the implant sites reached the level of significance for *P. gingivalis*, *P. micra*, and *F. nucleatum*. These three species have been linked to a higher risk of developing peri-implantitis [19, 25–27]. In conclusion, colonization of the submucosal peri-implant area is similar to the composition of the subgingival dentate microbiota. Smoking affects the colonization of implant-associated biofilms and may favor the periodontal pathogens *P. gingivalis*, *P. micra*, and *F. nucleatum*.
